# Polyvinylnorbornene Gas Separation Membranes

**DOI:** 10.3390/polym11040704

**Published:** 2019-04-17

**Authors:** Wouter Dujardin, Cédric Van Goethem, Julian A. Steele, Maarten Roeffaers, Ivo F. J. Vankelecom, Guy Koeckelberghs

**Affiliations:** 1 Laboratory for Polymer Synthesis, Department of Chemistry, KU Leuven, Celestijnenlaan 200F, B-3001 Heverlee, Belgium; wouter.dujardin@kuleuven.be; 2 Centre for Surface Chemistry and Catalysis, Department of Microbial and Molecular Systems, KU Leuven, Celestijnenlaan 200F, B-3001 Heverlee, Belgium; cedric.vangoethem@kuleuven.be (C.V.G.); julian.steele@kuleuven.be (J.A.S.); maarten.roeffaers@kuleuven.be (M.R.); ivo.vankelecom@kuleuven.be (I.F.J.V.)

**Keywords:** polynorbornene, addition polymerization, crosslinking, photoinitiator, TPO, gas separation, membrane, copolymerization, 5-vinyl-2-norbornene, free-standing membrane

## Abstract

Polynorbornenes are already used in a wide range of applications. They are also considered materials for polymer gas separation membranes because of their favorable thermal and chemical resistance, rigid backbone and varied chemistry. In this study, the use of 5-vinyl-2-norbornene (VNB), a new monomer in the field of gas separations, is investigated by synthesizing two series of polymers via a vinyl-addition polymerization. The first series investigates the influence of the VNB content on gas separation in a series of homo and copolymers with norbornene. The second series explores the influence of the crosslinking of polyvinylnorbornene (pVNB) on gas separation. The results indicate that while crosslinking had little effect, the gas separation performance could be fine-tuned by controlling the VNB content. As such, this work demonstrates an interesting way to significantly extend the fine-tuning possibilities of polynorbornenes for gas separations.

## 1. Introduction

Norbornene (NB, bicyclo[2.2.1]-2-heptene) and its many derivatives are used in a wide range of applications such as photoresists, adhesives and rubbers [1,2,3,4]. They can be polymerized in various ways depending on the desired properties of the end material [5,6,7]. If ring-opening metathesis polymerization (ROMP) is used, rubbery polynorbornenes with an unsaturated backbone are formed. A second possibility is a vinyl-addition polymerization which typically produces polynorbornenes with rigid saturated backbones with high glass transition temperature (*T*_g_) [8]. This variety of monomers and polymerization methods produces a wealth of different polynorbornenes which can be used for gas separation membranes [6,8,9,10,11]. Specifically, vinyl-addition polynorbornenes show a lot of promise as they have higher thermal stability, plasticization resistance and a rigid and sterically demanding bicyclic backbone [6,11]. This last property mimics the principles of polymers of intrinsic microporosity (PIMs) which have proven that high permeabilities could be obtained with soluble glassy polymers by having a rigid and contorted backbone [12,13,14].

**Figure 1:**
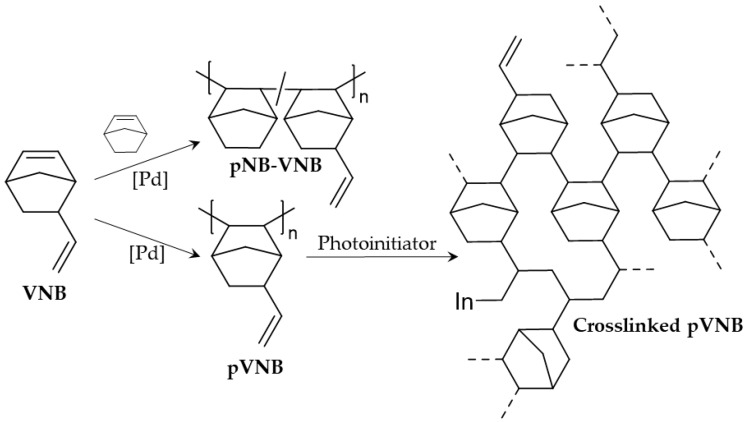
The structure of VNB, pVNB, copolymers pNB-VNB and crosslinked pVNB.

The gas separation properties of many vinyl-addition polynorbornenes have been intensively studied [6]. Polynorbornene with trimethylsilyl groups showed an extremely high CO_2_ permeability of 4350 Barrer but a low CO_2_/CH_4_ selectivity of 5.5 [15]. Recently, high CO_2_ permeabilities were achieved (1350 Barrer) with a CO_2_/N_2_ selectivity of 17 by using a triethylsiloxane pendant side group [9] and impressive BET surface areas (420–970 m^2^/g) were achieved with derivatized polynorbornenes [16]. From the wide variety of functionalized norbornene monomers that were used for gas separation membranes, 5-vinyl-2-norbornene (VNB) was still missing. This is surprising as this monomer presents possibilities of copolymerization with other norbornenes, post-polymerization functionalizations and crosslinking, significantly increasing application and fine-tuning options (Figure 1). In this manuscript, a first step is taken by studying the influence of the copolymerization with NB and the crosslinking with a photoinitiator on the gas separation performance.

First, a series of copolymers of NB and VNB was synthesized to study the influence of the exocyclic vinyl group on gas separation. Previous results have shown that the gas permeability is extremely dependent on the size of the side chain: Replacing hydrogen with a mere methyl group can result in a permeability increase from 34–49 to 81–396 Barrer [6,7,15,17]. Therefore, the VNB feed content in a series of polynorbornenes using NB and VNB was varied using 0% (only NB), 25%, 50%, 75% and 100% (only VNB). A Pd catalyst was used, capable of specifically targeting the cyclic double bond and leaving the exocyclic double bond intact. The resulting copolymer membranes were then tested for their gas separation performance.

Crosslinking is often used to improve gas separation performances by fine-tuning retention and permeance or by increasing plasticization resistance [18,19,20]. Therefore, a second series was developed involving the crosslinking of polyvinylnorbornene (pVNB) using its exocyclic vinyl group with a photoinitiator. It is known that polymers with unsaturated carbon bonds in their backbone, such as with ROMP polynorbornenes, are degraded by UV irradiation [21,22]. In contrast, vinyl-addition polynorbornenes with an unsaturated side group, such as 5-ethylidene-2-norbornene, are suitable for this type of crosslinking [21,22]. A well-known photoinitiator, diphenyl(2,4,6-trimethylbenzoyl)phosphine oxide (TPO), was chosen as photoinitiator as it offers a beneficial balance between photostability and photoreactivity [23]. This balance is important to control the degree of crosslinking. Various TPO loadings in the membrane were used to introduce varying degrees of crosslinking and their influence on the gas separation performance was assessed.

## 2. Materials and Methods

### 2.1. Materials

All solvents were purchased from Chem-Labs (Zedelgem, Belgium), VWR (Oud-Heverlee, Belgium) or Fisher Scientific (Merelbeke, Belgium). All reagents were purchased from Acros Organics (Geel, Belgium), AK Scientific (Union city, CA, USA), Sigma-Aldrich (Overijse, Belgium), Fischer Scientific (Merelbeke, Belgium) or J&K Scientific (Overpelt, Belgium). Toluene was dried by a solvent purification system MBRAUN SPS 800. Other solvents and reagents were used as received unless specified otherwise. NB was sublimed under reduced pressure. All reactions were done in oven-dried or flame-dried apparatus under nitrogen or argon atmosphere.

### 2.2. Characterization Methods

Proton nuclear magnetic resonance (^1^H NMR) spectra were recorded on a Bruker Ascend 400 MHz or a Bruker Avance II 600 MHz spectrometer. Infrared spectra were recorded on a Bruker Alpha FT-IR-spectrometer equipped with a Platinum ATR single reflection diamond ATR module. A 2 cm^−1^ resolution was used with 24 scans. Gel permeation chromatography (GPC) measurements were carried out on a Shimadzu 10A GPC system. The column was a PLgel 5 µm mixed-D type column which is kept at 35 °C. High-performance liquid chromatography (HPLC) grade tetrahydrofuran (THF) or chloroform were used as solvent with a flow rate of 1.0 mL/min. The detection system consisted of a differential refractometer. The GPC system was calibrated toward polystyrene standards purchased from Polymer Laboratories. The polymer samples were dissolved in the same solvent mixture as used for the GPC with a concentration of about 1 mg/ml. Once dissolved, the samples were filtered over a polytetrafluoroethylene (PTFE) filter with a pore size of 0.2 µm. The X-ray diffraction patterns were collected in transmission mode on a STOE Stadi P high-throughput powder diffraction apparatus equipped with a CuKα X-ray tube (λ = 1.5418 Å) and an image plate detector. The *T*_g_ of the polymers was recorded using differential scanning calorimetry (DSC). The measurements were performed with a TA Instruments Q2000-DSC. DSC samples were prepared by weighing 4–7 mg pieces of membrane in Tzero Aluminium Hermetic pans and calibration was done towards an empty pan. A heating rate of 10°/min was employed up to 340 °C under nitrogen flow, after which the sample was cooled to 25 °C at the same rate. The thermal degradation temperature and dryness of the membranes were tested by TGA under nitrogen atmosphere. A TA Instruments TGA-Q500 was used with 5 mg polymer membrane samples on a platinum pan. The samples were kept isothermal for 5 min at 50 °C after which they were heated at 20 °C/min up to 700 °C. The membrane cross-section microstructure was visualized by using a Jeol JSM-6010LV scanning electron microscope operated at 10 kV. The cross-sections were obtained by freeze-fracturing the samples in liquid nitrogen. All samples were sputter coated with Au/Pd for two times 30 s by a Jeol JFC-1300 autofine sputter coater in order to minimize sample charging during imaging. The membrane thickness was measured by a Mitutoyo Disk micrometer (369-511-30, ±4 µm). For UV induced crosslinking, a UV chamber with 3 LEDs (365 nm, output power 200 mW) was used. Raman scattering spectra were recorded using a home-built optical microscope (Ti−U, Nikon) in confocal mode. An air objective of 0.92 NA and 60× magnification was used to focus the 632.8 nm line of a He-Ne laser for Raman excitations. The optical power density was regulated using neutral density filters and a 650 nm long-pass filter was employed to filter Rayleigh scattering. Dispersion was achieved using a 600 g/mm, resulting in a spectral resolution of 0.5 cm^−1^.

### 2.3. Polymerization

#### 2.3.1. Polynorbornene (pNB)

Dichloromethane (DCM) was dried by refluxing with CaH_2_ and subsequent distillation. The solvent was then stored on molecular sieves. NB (940 mg, 10.0 mmol) was weighed in a flask and put under dry atmosphere. Next, DCM (4 mL) was added. After dissolution, Ni(C_6_F_5_)_2_(SbPh_3_)_2_ (10.8 mg, 10.0 µmol) was added. After 10 s, white precipitate formed. The mixture was then poured in 200 mL acetone and stirred for 1 h. Afterwards, the mixture was filtrated and the resulting white polymer was dried under reduced pressure at 60 °C.

Yield: 0.556 g of white fibers (58%).

#### 2.3.2. pNB-VNB-50 Using Ni(C_6_F_5_)_2_(SbPh_3_)_2_

NB (942 mg, 10.0 mmol) and 5-vinyl-2-norbornene (1.43 mL, 10.0 mmol) were dissolved in 3 mL dichloromethane. While stirring, Ni(C_6_F_5_)_2_(SbPh_3_)_2_ (21.6 mg, 20.0 µmol) was added and the flask was brought under dry atmosphere. Another 3 mL dichloromethane was added and the reaction was continued at 30 °C for overnight. Afterwards, the reaction mixture was poured in stirring methanol, forming white fibers. The fibers were collected by filtration over a filter and analyzed by GPC.

#### 2.3.3. pVNB Using Ni(C_6_F_5_)_2_(SbPh_3_)_2_

The same methodology was used as with pNB-VNB-50 using the same catalyst. 5-vinyl-2-norbornene (600 mg, 5.00 mmol) was polymerized with Ni(C_6_F_5_)_2_(SbPh_3_)_2_ (5.40 mg, 5.00 µmol).

#### 2.3.4. pNB-VNB-50 Using Pd_2_dba_3_/AgSbF_6_/PPh_3_

NB (1.84 g, 20.0 mmol) and 5-vinyl-2-norbornene (2.40 g, 20.0 mmol) were mixed, purged with N_2_ and sonicated. Toluene was purged with N_2_ and sonicated as well. Meanwhile, Pd_2_dba_3_ (9.20 mg, 10.0 µmol, 20.0 µmol of Pd), AgSbF_6_ (10.3 mg, 30.0 µmol) and PPh_3_ (5.30 mg, 20.0 µmol) were added to a flask and put under dry atmosphere. Toluene (4 mL) was added to the solids and the mixture was heated to 70 °C. The norbornene mixture was added to the catalyst solution using a transfer needle. After 15 h, a brown gel-like solid had formed and no free solvent was visible. The solid was transferred to a beaker and toluene (70 mL) was added, dissolving the solid. The polymer was then measured on GPC.

#### 2.3.5. pVNB Using Pd_2_dba_3_/AgSbF_6_/PPh_3_

The same methodology was used as with pNB-VNB-50 using the same catalyst. The 5-vinyl-2-norbornene (36.0 g, 300 mmol) was polymerized with Pd_2_dba_3_ (27.5 mg, 30.0 µmol, 60.0 µmol of Pd), AgSbF_6_ (24.8 mg, 72.0 µmol) and PPh_3_ (15.7 mg, 60.0 µmol) using 30 mL toluene.

#### 2.3.6. pVNB Using Pd_2_dba_3_/TTPB/PCy_3_

In a 25 mL flask, Pd_2_dba_3_ (9.10 mg, 10.0 µmol), triphenylcarbenium tetrakis(pentafluoro)borate (TTPB, 18.4 mg, 20.0 µmol), PCy_3_ (5.60 mg, 20.0 µmol) and toluene (20 mL) were added. This solution was brought under inert atmosphere and stirred for 5 min. In a 50 mL flask under inert atmosphere, 5-vinyl-2-norbornene (1.20 g, 10.0 mmol) was added and stirred. 10 mL of the catalyst solution and 20 mL of toluene was added to the monomer. The reaction temperature was set at 40 °C and the polymerization was continued for 5 h. Afterwards, the solution was poured in methanol, yielding white fibers. The fibers were collected by vacuum filtration over a filter and dried under vacuum at room temperature overnight.

Yield: 1.12 g of white fibers (93%).

#### 2.3.7. pNB-VNB Copolymers Using Pd_2_dba_3_/TTPB/PCy_3_

The copolymers were made using the same modus as pVNB. The specific monomer amounts and resulting yields are summed up in Table 1.

### 2.4. Membrane Preparation

The membranes of pNB, pNB-VNB-25, pNB-VNB-50, pNB-VNB-75 and pVNB were prepared by dissolving 400 mg of polymer in 20 mL of chloroform. After 5 days of stirring, the solutions were filtrated and the resulting clear solutions were poured in Teflon Petri dishes (4.7 cm diameter). These Petri dishes were covered with a blocked funnel and put in a nitrogen glove bag. After at least 40 days of drying, they were used for gas separation and membrane characterization tests.

The crosslinked membranes (pVNB-0%, pVNB-0.13%, pVNB-1.3%, pVNB-33%, pVNB-65%) were prepared by adding 6.75 mL of the polymer solution containing 193 mg pVNB to a flask. Then, the respective amounts of TPO were added and the total volume was diluted to 14 mL. These solutions were filtered and subsequently poured in Teflon Petri dishes (4.7 cm diameter), which were covered with a blocked funnel, shielded from light and put in a nitrogen glove bag. After 5 days of drying, they were put in a UV chamber at 365 nm for 1 h to crosslink the membranes. The inner temperature of the UV chamber did not exceed 50 °C, making thermal crosslinking unlikely. After crosslinking, the membranes were soaked in methanol for 5 h to remove excess TPO and its degradation products. The membranes were collected from methanol and dipped dry with a towel. Next, they were dried under vacuum at 70 °C for 12 h. The gas separation tests were done within 24 h after drying.

### 2.5. Gas Separation Measurements

The gas separation properties were assessed using high-throughput gas separation equipment [24]. The experiments were performed at 35 °C, feed pressure was set at 2.5 bar and the permeate side was kept under vacuum. For the mixed-gas tests, a 50/50 mixture was used at 5 bar. The permeate gas composition was determined by a compact gas chromatograph (Interscience).

The mixed-gas selectivity was calculated by Equation (1) with $x_{i}$ and $y_{i}$ the mole fractions of gas i in, respectively, the up and downstream:

(1) $$\alpha_{ij}=\frac{y_{i}/y_{j}}{x_{i}/x_{j}}$$

The mixed and single-gas permeability of the membrane was determined by recording the increase in gas pressure in a fixed volume cylinder using Equation (2):

(2) $$P_{i}=\frac{273\times 10^{6}}{760}\frac{y_{i}V}{AT\;\left(\frac{76}{14.7}\right)x_{i}P_{2}}\frac{dP}{dt}$$

With V the downstream volume in cm^3^ and dP/dt is the pressure rate. A refers to the membrane area in cm^2^, T is the cell temperature in Kelvin, and $P_{2}$ is the feed pressure in Psi. 

## 3. Results

### 3.1. Polynorbornenes with Increasing VNB Content

#### 3.1.1. Polymerization and Characterization

Polynorbornene (pNB) was easily synthesized using the catalyst Ni(C_6_F_5_)_2_(SbPh_3_)_2_ [10] (Table S1, entry 1). However, synthesizing polymers made with 5-vinyl-2-norbornene proved to be more difficult. Neither Ni(C_6_F_5_)_2_(SbPh_3_)_2_ or Pd_2_dba_3_/AgSbF_6_/PPh_3_ [25] were capable of obtaining large molecular weights with a monomer mixture of NB and VNB (ratio 50:50) or with only VNB (Table S1, respectively entries 2-3 and 5-6). VNB is known to be challenging to polymerize as the exocyclic vinyl group can act as a reactive site for chain transfer [26] or it can slow the catalyst down by sterical hindrance or coordination [26,27]. Since large molecular masses are required to obtain mechanically stable membranes, another catalyst system was employed using Pd_2_dba_3_/TTPB/PCy_3_ [26] (Table S1, entries 4 and 7), resulting in number average molecular weights above 167 kg/mol. This proved to be sufficient for producing mechanically stable free-standing membranes. Therefore, with this catalyst system, a series of polynorbornenes with varying VNB content were synthesized. During workup, aliquots were taken for GPC analysis in THF (Table 2, Figure S1). The aliquots did not precipitate in THF, but had difficulty passing through a 0.2 µm filter. Possibly, the higher molar mass chains were retained, resulting in an underestimation of the real molar masses. Still, high molar masses were obtained for all polymers. 

**Figure 2:**
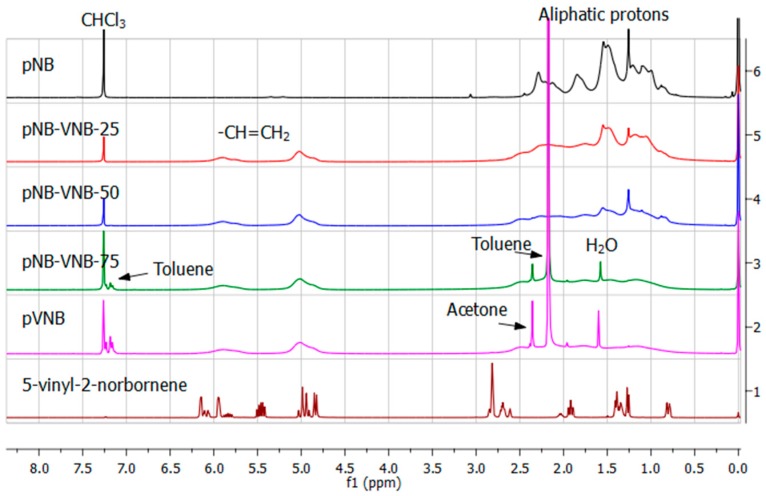
^1^H NMR spectra, from top to bottom, of pNB, pNB-VNB-25, pNB-VNB-50, pNB-VNB-75, pVNB and the monomer VNB. Residual solvent signals from work up are marked in spectra of less soluble pNB-VNB-75 and pVNB.

The solubility of the polymers after drying proved to be challenging. pNB, pNB-VNB-25 and pNB-VNB-50 were soluble in chloroform. pNB-VNB-75 and pVNB were stored in solution as they would become insoluble after drying. As a consequence, not all solvent could be removed, resulting in large residual solvent signals in the NMR spectra (Figure 2). The ^1^H NMR spectra showed that the polymers were monomer free as no sharp correlating monomer signals are present in the polymer spectra. Also, by equalizing the vinyl proton signal intensity (4.5–6.5 ppm), a gradual decrease in aliphatic signals (0.5–2.5) is visible. This indicates that an increasing amount of VNB to NB is built in, which is in accordance with the monomer composition. The integration of the signals indicate that for pNB-VNB-25 and for pNB-VNB-50 respectively about 31% and 55% VNB was built in (Figures S10 and S11). These values may be slightly overestimated due to the presence of solvent signals in the aliphatic region (e.g., water).

**Figure 3:**
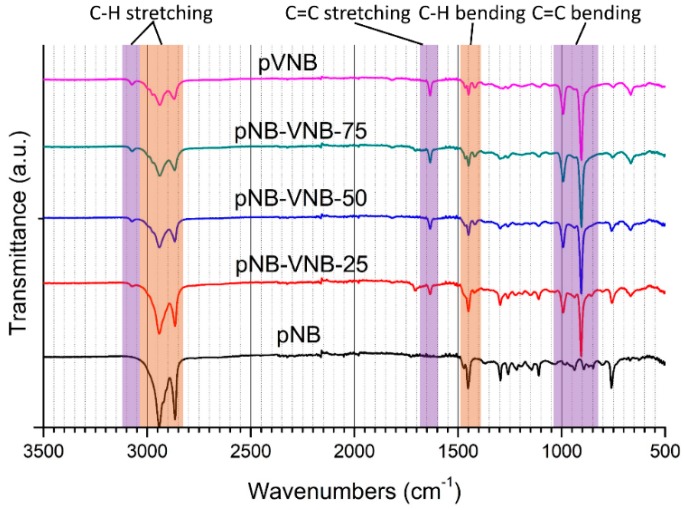
FT-IR of polynorbornene membranes with various amounts of VNB built in. Purple regions are unique for alkenes, while orange regions are related to saturated C–H bonds.

The free-standing membranes were analyzed by Fourier-transform infrared spectroscopy (FT-IR) (Figure 3). Analyzing the spectra with increase in VNB content, it is clear that the purple regions, related to the unsaturated C=C and C–H bonds, increase in intensity while the orange regions, related to the saturated C–H bonds, decrease in intensity. This confirms the conclusion from the NMR spectra: NB-VNB copolymers can be synthesized by controlling the VNB content in the polymerization feed. 

**Figure 4:**
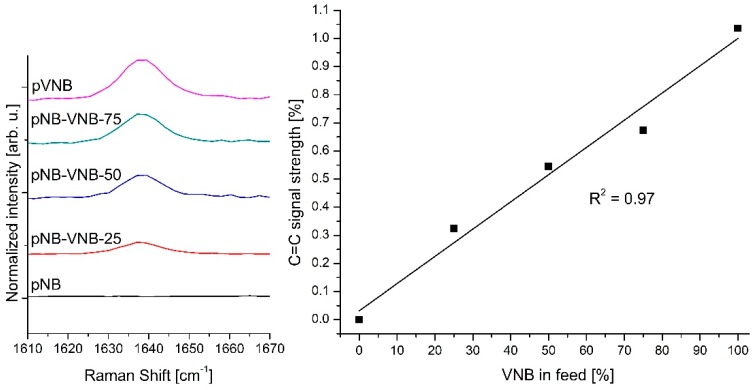
Raman analysis of the membranes. Left: A comparison of the Raman signals recorded over the C=C vibrational band. To facilitate the comparison and analysis leading these data, the C=C band intensity has been normalized relative to their common CH_x_ band at 2950 cm^−1^ (Figure S7). Right: The C=C signal intensity at 1637 cm^−1^ is plotted against the VNB content in the monomer feed.

Raman, which is highly sensitive to the C=C stretching vibration, allow for a deeper analysis of the VNB content in the polymer membranes, by analyzing the relative changes in the C=C signal at 1637 cm^−1^. For this, the Raman spectra were recorded across different VNB feed concentrations using their common and invariant CH_x_ signatures just below 3000 cm^−1^ and were subsequently normalized. As such, the relative intensity of the C=C signal in the material can be quantified and compared directly. This analysis is presented in Figure 4, which exhibits a near perfect correlation between VNB content in the monomer feed and the C=C signal strength at 1637 cm^−1^. This correlation is in accordance with the VNB content analysis by NMR signal integration for pNB-VNB-25 and pNB-VNB-50. Such an approach may facilitate a direct means of analyzing the VNB content when such details are not known. 

**Figure 5:**
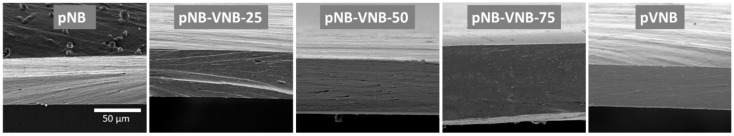
SEM pictures of the cross-sections of the polynorbornene membranes. All membranes were imaged using the same magnification (×500). Larger images and images with higher magnification can be found in the supporting information.

Scanning Electron Microscopy (SEM) was used to view the cross-sections of the membranes (Figure 5). All membrane cross-sections show homogeneous dense films.

Thermogravimetric analysis (TGA) showed no discernable pattern with increasing VNB content (Figure S3). The degradation temperature of pVNB at 5% mass loss was 350 °C, indicating high thermal stability. No *T*_g_ was detected under 340 °C, confirming the rigidity of the polynorbornenes (Figure S5).

**Figure 6:**
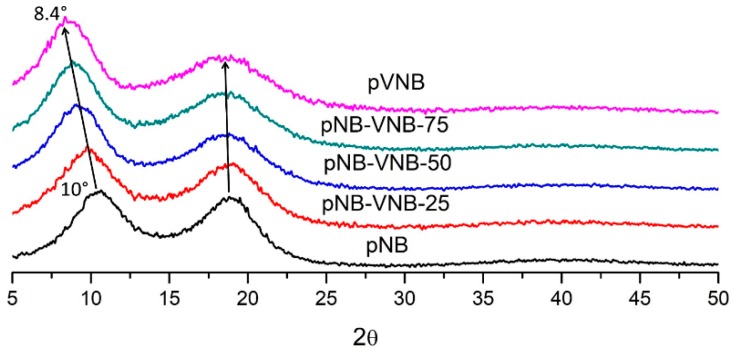
Normalized WAXS spectra of polynorbornenes with increasing VNB content.

The wide-angle X-ray scattering (WAXS) pattern of the polynorbornenes included two broad peaks, indicating that the polymers are completely amorphous (Figure 6). It is generally accepted that the peak at higher 2θ correlates with intrasegmental interactions while the peak at lower 2θ correlates with intersegmental interactions [15,28,29]. Three interesting trends are visible with an increase in exocyclic vinyl group presence in the polymers. First, the intrasegmental peak broadens at higher 2θ. This can be explained by the increasing presence of the exocyclic vinyl groups which may be capable of increasing the amorphous character of the intrasegmental packing. Second, the intersegmental peak shifts from 10° to 8.4°. This corresponds, using Bragg’s law, with a *d*-spacing shift from 8.8 to 10.5 Å, indicating that the increase in exocyclic vinyl group presence considerably increases intersegmental *d*-spacing. Third, relatively increasing scattering intensities of the low 2θ peak with increase in VNB content indicates that more numerous intersegmental scattering events took place compared to the intrasegmental scattering. Therefore, based on these observations, an increase in exocyclic vinyl group content seems to benefit larger intersegmental spacing. Comparing this data with other addition type polynorbornenes with different side groups, it is clear that the X-ray scattering data for pNB is similar to previously published pNB X-ray data (Table 3) [17]. Also, the intersegmental *d*-spacing of pVNB is situated between the intersegmental *d*-spacings of polynorbornene with a methyl group and with a butyl group, further confirming the trend that intersegmental *d*-spacing increases with increasing side-group bulkiness [28,30].

#### 3.1.2. Gas Separations

**Figure 7:**
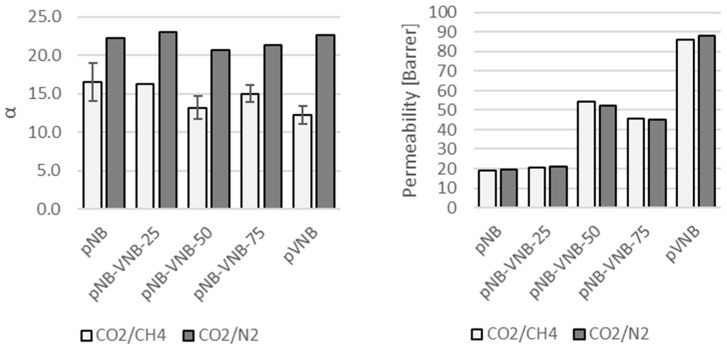
(**Left**) Mixed-gas selectivity and (**right**) mixed-gas permeability for CO_2_/CH_4_ and CO_2_/N_2_ for the series of polynorbornenes with increasing VNB content.

First, the membranes were tested for CO_2_/CH_4_ and CO_2_/N_2_ separations using a mixed-gas feed (Figure 7) to investigate the influence of the exocyclic vinyl group. The permeability quadrupled for both gas pairs with increasing VNB content, while α_CO2/CH4_ declined slightly and α_CO2/N2_ remained largely constant, even though trends hardly exceed the experimental error. Single-gas permeabilities were collected as well to easily compare them with other published data (Table 4). Unfortunately, the membrane pNB-VNB-25 had become defective and was left out of the analysis. For the permeabilities, a trend is visible confirming the previous mixed-gas permeabilities: By increasing the VNB content, the permeability increases. pVNB even achieved a CO_2_ permeability of 104 Barrer. This is also in line with the WAXS trend which shows that a larger VNB content resulted in larger *d*-spacings. The permeabilities obtained in this work for pNB are close to those previously published [15]. The permeabilities of pVNB are close to the one of the polynorbornenes with a methyl side group [28]. The ratio of two single-gas permeabilities gives the ideal selectivity of that gas pair. The ideal selectivity differs from mixed-gas selectivity in the sense that it allows for a more direct comparison with other single gas measurements and the resulting ideal selectivities, but it does not take into account any interactions between the mixture components as is the case with mixed-gas selectivity. These ideal selectivities show a slight decrease with increase in VNB content (Table 5). In the case of H_2_/CO_2_, this even results in a reverse selectivity for pVNB.

### 3.2. Crosslinking of pVNB

#### 3.2.1. Polymerization, Crosslinking and Characterization

**Figure 8:**
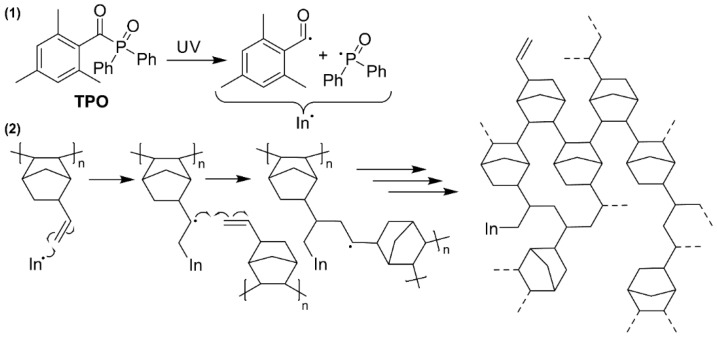
(**1**) TPO produces two initiator (In) radicals by exposure to UV light. (**2**) The initiator radicals start a radical polymerization with the exocyclic vinyl groups, thereby crosslinking pVNB.

The exocyclic vinyl group offers possibilities such as post-polymerization functionalization and crosslinking. In this section, the influence of crosslinking on the gas separation properties such as mixed-gas selectivity and permeability and CO_2_ plasticization is investigated. Therefore, five crosslinked polymer membranes were prepared from pVNB with the following TPO loadings: 0 mol % (pVNB-0%), 0.13 mol % (pVNB-0.13%), 1.3 mo l% (pVNB-1.3%), 33% (pVNB-33%) and 65% (pVNB-65%). These loadings were chosen as they cover a broad range, enabling the detection of interesting effects both in the lower and higher loading region. The crosslinking mechanism is explained in Figure 8.

**Figure 9:**
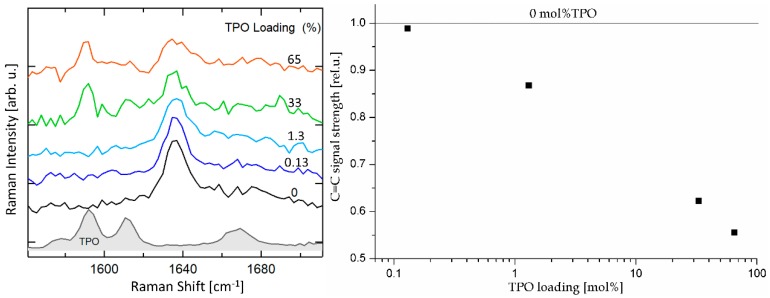
Raman analysis of the membranes. Left: Normalized Raman spectra of pVNB with varying TPO content and of UV treated TPO. The normalization was done similarly as for Figure 4. The CH_x_ band region is shown in Figure S8. Right: C=C signal strength of Raman spectra plotted against the TPO content.

The extent of crosslinking was first assessed by FT-IR (Figure S2) where no measurable difference in C=C stretching or bending signals were found. In contrast, Raman clearly showed a decrease in C=C signal at 1637 cm^−1^ with an increase in TPO loading (Figure 9). This confirms that the extent of crosslinking can be controlled by varying the TPO loading. However, no full conversion could be obtained as even with 65 mol % TPO, only about 55% of vinyl groups were converted.

Crosslinking surprisingly had a slightly detrimental effect on the thermal stability (Figure S4). As double carbon bonds are converted into single carbon bonds, a higher thermal stability, close to that of pNB would be expected. This is probably caused by the lower thermal resistance of TPO, possibly still present in the membrane [31]. Yet, no clear trend is visible within the membranes with higher TPO loading. Normally, as crosslinking increases chain rigidity, a higher *T*_g_ is expected for crosslinked polymers. No *T*_g_ was measured up to 250 °C using DSC (Figure S6). Higher temperatures were not feasible due to the decreased thermal resistance of the membranes with higher TPO loading. 

**Figure 10:**
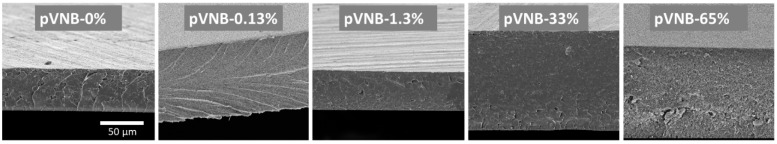
SEM pictures of the cross-sections of the polynorbornene membranes.

SEM was used to view the cross-sections of the polymer membranes, showing homogeneous dense films (Figure 10).

#### 3.2.2. Gas Separations

Defect-free membranes were obtained from pVNB-0%, pVNB-1.3%, pVNB-33% and pVNB-65%. Mixed-gas CO_2_/CH_4_ selectivity and permeability were measured to assess the influence of the crosslinking on the gas separation (Table 6). Overall, low to modest selectivities were obtained with no particular trend. The permeability declines with increasing TPO loading. This can be due to the presence of crosslinks, tightening the polymer network and, therefore, decreasing the size of the free volumes, resulting in lower permeability. Additionally, the presence of excess TPO and its degradation products can fill up the free volume, which also results in lower permeability. Overall, these results demonstrate that crosslinking pVNB has little benefit to its gas separation performance. Nevertheless, in combination with high-permeability polynorbornenes, such as those with a trialkylsilyl side chain, copolymerization with VNB and subsequent crosslinking might improve stability, possibly leading to high-performance gas separation membranes.

### 3.3. Robeson Plot

Robeson plots are used to put the gas separation performance of pVNB and its derivatives in perspective to other polynorbornenes and state-of-the-art polymers for the gas pairs CO_2_/CH_4_ and CO_2_/N_2_. Robeson plots are ubiquitous to compare gas separation performances of polymer membranes to each other and to the ‘upper bound’, a line visualizing the trade-off between permeability and selectivity [32,33]. Typically, only single-gas permeability and ideal selectivity are used for a Robeson plot. However, both single and mixed-gas data were collected in this research. While single-gas data collection has the advantage to quickly scan through multiple gas pairs, it lacks the capability to assess the interaction between the different gas molecules. To calculate the permeability of 1 species from a mixed-gas experiment, Equation (3) was used, while the interaction between the different gas molecules was assumed negligible.

(3) Pi=Pmixed(1+1αmixed)×xi

**Figure 11:**
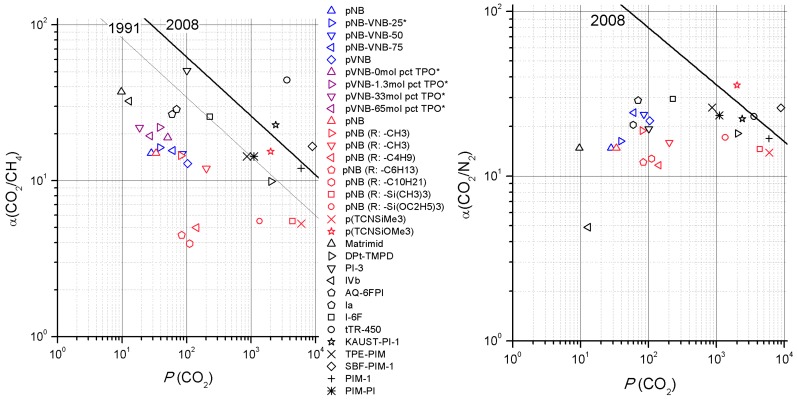
(**Left**) CO_2_/CH_4_ and (**Right**) CO_2_/N_2_ Robeson plots with polynorbornenes with increasing VNB content (blue), pVNB with increasing TPO loading (purple), other polynorbornenes (pink) and polyimides, PIMs and thermally rearranged polymers (black). * Single-gas P_CO2_ data [Barrer] obtained using Equation (3). Data obtained from [7,9,15,28,34,35,36,37,38,39,40,41,42,43,44]. Upper bound data was taken from [33].

$P_{mixed}$ is the mixed-gas permeability, $\alpha_{mixed}$ mixed-gas selectivity and $x_{i}$ the mole fraction of gas i in the upstream. The Robeson plots (Figure 11) show that selectivity of the crosslinked polynorbornenes is a little higher and their permeability somewhat lower than the polynorbornenes with increasing VNB content. This latter series’ performance is close to the performance of one of the two polynorbornenes with a methyl side chain. Compared to the other polynorbornene derivatives, overall better selectivities are obtained with largely comparable permeabilities. In comparison with state-of-the-art polymers (black), relatively modest performances are obtained by using polynorbornenes.

## 4. Conclusions

The use of VNB was introduced as a new building block for polynorbornenes gas separation membranes. Two series of membranes were prepared: A series varying the VNB content from pure pNB to pure pVNB and a series with varying extent of pVNB crosslinking. With increasing VNB content, higher permeabilities and lower selectivities were obtained, which was in agreement with increasing *d*-spacings, as measured by WAXS. With an increasing degree of crosslinking, the selectivity did not change significantly, while the permeability decreased. Overall, compared to other polynorbornenes, similar permeabilities with higher selectivities were obtained. Compared to state-of-the-art polymers (e.g., PIMs), the gas separation performance is still modest. As this polymer class has the potential to provide chemically and thermally robust polymers which can be easily synthesized, further research into new building blocks and methods of fine-tuning the polymer properties should be used in order to improve their basic gas separation performance. 

## Figures and Tables

**Table 1:** Polymerization parameters for the copolymers using NB and VNB.

	NB		VNB		Yield	
pNB-VNB-25	706 mg	7.50 mmol	300 mg	2.50 mmol	754 mg	75%
pNB-VNB-50	471 mg	5.00 mmol	601 mg	5.00 mmol	836 mg	78%
pNB-VNB-75	235 mg	2.50 mmol	901 mg	7.50 mmol	935 mg	82%

**Table 2:** Reaction and GPC data for polynorbornene series with increasing exocyclic vinyl group presence. Calibrated using polystyrene standards.

Polymer	Catalyst	$\frac{\mathrm{Monomer}}{\mathrm{Catalyst}}$	Yield	${\bar{M}}_{n}(\mathrm{kg}/\mathrm{mol})$	${\bar{M}}_{w}(\mathrm{kg}/\mathrm{mol})$	Đ
pNB ^b^	Ni(C_6_F_5_)_2_(SbPh_3_)_2_	1000	58%	84	277	3.3
pNB-VNB-25 ^a^	Pd_2_dba_3_/TTPB/PCy_3_	1000	75%	121	263	2.2
pNB-VNB-50 ^a^	Pd_2_dba_3_/TTPB/PCy_3_	1000	78%	167	440	2.6
pNB-VNB-75 ^a^	Pd_2_dba_3_/TTPB/PCy_3_	1000	82%	104	198	1.9
pVNB ^a^	Pd_2_dba_3_/TTPB/PCy_3_	1000	93%	349	471	1.4

^a^ Data obtained in THF, ^b^ data obtained in chloroform.

**Table 3:** WAXS data for addition type polynorbornenes with different side groups (R).

Polynorbornene	2θ [°]		*d*-Spacing [Å]		Ref
	Low Angle	High Angle	Low Angle	High Angle	
pNB	10	18.5	8.8	4.7	[17]
pNB	10	18.8	8.8	4.7	this work
pNB (R: –CH_3_)	9.5	18.2	9.3	4.9	[28]
pVNB	8.4	18.6	10.5	4.8	this work
pNB (R: n–C_4_H_9_)	7.2	18.8	12.2	4.7	[30]
pNB (R: –Si(CH_3_)_3_)	6.5	15.5	13.6	5.7	[15]
pNB (R: –n–C_6_H_13_)	6.6	19.2	13.5	4.6	[30]

**Table 4:** Single-gas permeability data of polynorbornenes with increasing VNB content after 100 days of aging and other polynorbornenes with different side groups (R) performances.

Polynorbornenes	Catalyst	Single-Gas Permeability [Barrer]				Ref.
		CH_4_	CO_2_	N_2_	H_2_	
pNB	Ni ^a^	2.0 ± 0.7	28.2 ± 5.3	1.9 ± 0.5	59.3 ± 10	This work
pNB-VNB-50	Pd ^b^	5.7	85.3	3.6	89.6	This work
pNB-VNB-75	Pd ^b^	3.9 ± 0.1	60.8 ± 2.1	2.5 ± 0.1	64.8 ± 2.3	This work
pVNB	Pd ^b^	8.3 ± 1.4	104.3 ± 3.2	4.8 ± 1.0	88.7 ± 5.4	This work
pNB	Ni ^c^	2.6	33.6	1.5	41.5	[15]
pNB (R: –CH_3_)	Ni ^d^	5.6	81.1	4.3	-	[28]
pNB (R: –CH_3_)	Pd ^d^	16.9	202.1	12.6	-	[28]
pNB (R: –Si(CH_3_)_3_)	Ni ^d^	790	4350	297	1680	[15]

^a^ Ni(C_6_F_5_)_2_(SbPh_3_)_2_, ^b^ Pd_2_dba_3_/TTPB/PCy_3_, ^c^ Ni [(Nph)_2_Ni]-methylaluminoxane, ^d^ no further information on the catalyst was given.

**Table 5:** Ideal selectivity of polynorbornenes with increasing VNB content.

Polynorbornenes	Single-Gas Selectivity				
	CO_2_/CH_4_	CO_2_/N_2_	H_2_/CO_2_	H_2_/CH_4_	H_2_/N_2_
pNB	15.0 ± 2.9	15.5 ± 1.3	2.1 ± 0.0	31.7 ± 6.5	32.7 ± 3.1
pNB-VNB-50	14.9	23.4	1.1	15.7	24.6
pNB-VNB-75	15.6 ± 0.1	24.2 ± 0.1	1.1 ± 0.0	16.6 ± 0.1	25.8 ± 0.1
pVNB	12.9 ± 1.6	22.4 ± 3.5	0.9 ± 0.0	10.9 ± 1.1	18.9 ± 2.5

**Table 6:** Mixed-gas properties of pVNB crosslinked with different TPO loadings.

pVNB with TPO Loading	P_CO2/CH4_ [Barrer]	α_CO2/CH4_
pVNB-0%	26.8 ± 3.3	18.9 ± 1.2
pVNB-1.3%	20.0	22.0
pVNB-33%	9.8 ± 0.4	21.9 ± 0.2
pVNB-65%	14.3 ± 1.7	19.4 ± 3.5

## Supplementary Materials

The following are available online at https://www.mdpi.com/2073-4360/11/4/704/s1, Table S1: GPC data of homopolymers pNB and pVNB and copolymers pNB-VNB-50, prepared by different catalyst systems. Figure S1: GPC spectra of polynorbornenes with increasing vinyl content. Figure S2: FT-IR of pVNB with increasing TPO loading. ‘TPO’ and ‘TPO - UV’ are, respectively, unexposed and exposed TPO to UV light. Figure S3: TGA thermogram of polynorbornenes membranes with increasing VNB content. Figure S4: TGA thermogram of crosslinked polyvinylnorborne membranes. Figure S5: DSC of polynorbornenes with increasing VNB content. Figure S6: DSC of polynorbornenes with increasing TPO loading. Figure S7: Comparison of Raman spectra recorded over the CH_x_ stretching region, for rising VNB in monomer feed. These spectra have been normalized relative to their common band at 2950 cm^−1^, which does not evolve with changing VNB concentration, allowing for the C=C band analysis shown in Figure 4 of the main article. Figure S8: Comparison of Raman spectra recorded over the CH_x_ stretching region, for increasing TPO content in the pVNB membranes. These spectra have been normalized relative to their common band at 2950 cm^−1^, which does not evolve with changing VNB concentration, allowing for the C=C band analysis shown in Figure 9 of the main article. Figure S9: SEM images of polynorbornene copolymers with increasing VNB content. Figure S10: _1_H NMR spectrum of pNB-VNB-25. (400 MHz, CDCl_3_). Figure S11. ^1^H NMR spectrum of pNB-VNB-50. (400 MHz, CDCl_3_).

## References

[1] Sumitomo Bakelite Co., LTD Promerus Introduction to Materials & Applications. https://www.promerus.com/wp-content/uploads/2017/10/Promerus-Materials-and-Applications-100317-v2.pdf.

[2] Smith S., Paudel L., Cyrus C., Burgoon H., Fujita K., Thoresen J., Thomas K., Langsdorf L., Rhodes L.F. (2018). Sugar-Functional Vinyl Addition Poly(norbornene)–Photopatternable Poly(norbornenyl gluconamide) Compositions Developed with Water. ACS Omega.

[3] Osokin Y.G. (2007). Vinylnorbornene: Preparation, chemical transformations, and use in organic synthesis and polymer chemistry. Vinylnorbornene synthesis and isomerization to ethylidenenorbornene (Review). Pet. Chem..

[4] Mol J.C. (2004). Industrial applications of olefin metathesis. J. Mol. Catal. A Chem..

[5] Finkelshtein E., Gringolts M., Bermeshev M., Chapala P., Yampolskii Y., Finkelshtein E. (2017). Membrane Materials for Gas and Vapor Separation.

[6] Yampolskii Y. (2010). Norbornene Polymers as Materials for Membrane Gas Separation. Comprehensive Membrane Science and Engineering.

[7] Dorkenoo K.D., Pfromm P.H., Rezac M.E. (1998). Gas transport properties of a series of high Tg polynorbornenes with aliphatic pendant groups. J. Polym. Sci. Part B Polym. Phys..

[8] Bermeshev M.V., Chapala P.P. (2018). Addition polymerization of functionalized norbornenes as a powerful tool for assembling molecular moieties of new polymers with versatile properties. Prog. Polym. Sci..

[9] Belov N., Nikiforov R., Starannikova L., Gmernicki K.R., Maroon C.R., Long B.K., Shantarovich V., Yampolskii Y. (2017). A detailed investigation into the gas permeation properties of addition-type poly (5-triethoxysilyl-2-norbornene). Eur. Polym. J..

[10] Gmernicki K.R., Hong E., Maroon C.R., Mahurin S.M., Sokolov A.P., Saito T., Long B.K. (2016). Accessing Siloxane Functionalized Polynorbornenes via Vinyl-Addition Polymerization for CO_2_ Separation Membranes. ACS Macro Lett..

[11] Finkelshtein E.S., Bermeshev M.V., Gringolts M.L., Starannikova L.E., Yampolskii Y.P. (2011). Substituted polynorbornenes as promising materials for gas separation membranes. Russ. Chem. Rev..

[12] Budd P.M., Ghanem B.S., Makhseed S., McKeown N.B., Msayib K.J., Tattershall C.E. (2004). Polymers of intrinsic microporosity (PIMs): Robust, solution-processable, organic nanoporous materials. Chem. Commun..

[13] Carta M., Malpass-Evans R., Croad M., Rogan Y., Lee M., Rose I., McKeown N.B. (2014). The synthesis of microporous polymers using Tröger’s base formation. Polym. Chem..

[14] Ghanem B.S., McKeown N.B., Budd P.M., Selbie J.D., Fritsch D. (2008). High-performance membranes from polyimides with intrinsic microporosity. Adv. Mater..

[15] Finkelshtein E.S., Makovetskii K.L., Gringolts M.L., Rogan Y.V., Golenko T.G., Starannikova L.E., Yampolskii Y.P., Shantarovich V.P., Suzuki T. (2006). Addition-Type Polynorbornenes with Si(CH_3_)_3_ Side Groups: Synthesis, Gas Permeability, and Free Volume. Macromolecules.

[16] Alentiev D., Dzhaparidze D., Gavrilova N., Shantarovich V., Kiseleva E., Topchiy M., Asachenko A., Gribanov P., Nechaev M., Legkov S. (2018). Microporous Materials Based on Norbornadiene-Based Cross-Linked Polymers. Polymers (Basel).

[17] Zhao C.T., Do Rosário Ribeiro M., De Pinho M.N., Subrahmanyam V.S., Gil C.L., De Lima A.P. (2001). Structural characteristics and gas permeation properties of polynorbornenes with retained bicyclic structure. Polymer (Guildf).

[18] Staudt-Bickel C., Koros W. (1999). Improvement of CO_2_/CH_4_ separation characteristics of polyimides by chemical crosslinking. J. Membr. Sci..

[19] Vanherck K., Koeckelberghs G., Vankelecom I.F.J. (2013). Crosslinking polyimides for membrane applications: A review. Prog. Polym. Sci..

[20] Ma C., Koros W.J. (2013). High-performance ester-crosslinked hollow fiber membranes for natural gas separations. J. Membr. Sci..

[21] Hilborn J., Rånby B. (1988). Photocrosslinking of EPDM Elastomers. A New Method for Rapid Curing of Elastomer Coatings at Room Temperature. Rubber Chem. Technol..

[22] Rånby B. (1998). Photochemical modification of polymers-photocrosslinking, surface photografting, and lamination. Polym. Eng. Sci..

[23] Rundlett B. Photoinitiator Selection. https://www.radtech.org/proceedings/2012/papers/end-user-presentations/LED/DSM_Rundlett_LED.pdf.

[24] Khan A.L., Basu S., Cano-odena A., Vankelecom I.F.J. (2010). Novel high throughput equipment for membrane-based gas separations. J. Membr. Sci..

[25] Commarieu B., Potier J., Compaore M., Dessureault S., Goodall B.L., Li X., Claverie J.P. (2016). Ultrahigh TgEpoxy Thermosets Based on Insertion Polynorbornenes. Macromolecules.

[26] Saito T., Wakatsuki Y. (2012). Addition polymerization of norbornene, 5-vinyl-2-norbornene and 2-methoxycarbonyl-5-norbornene with a catalyst based on a palladium(0) precursor complex. Polymer (Guildf).

[27] Funk J.K., Andes C.E., Sen A. (2004). Addition Polymerization of Functionalized Norbornenes: The Effect of Size, Stereochemistry, and Coordinating Ability of the Substituent. Organometallics.

[28] Wilks B.R., Chung W.J., Ludovice P.J., Rezac M.R., Meakin P., Hill A.J. (2003). Impact of average free-volume element size on transport in stereoisomers of polynorbornene. I. Properties at 35 °C. J. Polym. Sci. Part B Polym. Phys..

[29] Ahmed S. (1998). Stereochemical Structure-Property Relationships in Polynorbornene from Simulation. Ph.D. Thesis.

[30] Wilks B.R., Chung W.J., Ludovice P.J., Rezac M.E., Meakin P., Hill A.J. (2006). Structural and free-volume analysis for alkyl-substituted palladium-catalyzed poly(norbornene): A combined experimental and Monte Carlo investigation. J. Polym. Sci. Part B Polym. Phys..

[31] Ciba Speciality Chemicals Photoinitiators for UV Curing. https://people.rit.edu/deeemc/reference_13/Imprint/PhotoinitiatorsforUVcuring.pdf.

[32] Robeson L.M. (1991). Correlation of separation factor versus permeability for polymeric membranes. J. Membr. Sci..

[33] Robeson L.M. (2008). The upper bound revisited. J. Membr. Sci..

[34] Sulub-Sulub R., Loría-Bastarrachea M.I., Vázquez-Torres H., Santiago-García J.L., Aguilar-Vega M. (2018). Highly permeable polyimide membranes with a structural pyrene containing tert-butyl groups: Synthesis, characterization and gas transport. J. Membr. Sci..

[35] Dutta A., Bisoi S., Mukherjee R., Chatterjee R., Das R.K., Banerjee S. (2018). Soluble polyimides with propeller shape triphenyl core for membrane based gas separation. J. Appl. Polym. Sci..

[36] Yen H., Guo S., Yeh J., Liou G.-S. (2011). Triphenylamine-based polyimides with trimethyl substituents for gas separation membrane and electrochromic applications. J. Polym. Sci. Part A Polym. Chem..

[37] Hu Y.-C., Chen C.-J., Yen H.-J., Lin K.-Y., Yeh J.-M., Chen W.-C., Liou G.-S. (2012). Novel triphenylamine-containing ambipolar polyimides with pendant anthraquinone moiety for polymeric memory device, electrochromic and gas separation applications. J. Mater. Chem..

[38] Mao H., Zhang S. (2014). Synthesis, characterization and gas transport properties of novel poly(amine-imide)s containing tetraphenylmethane pendant groups. J. Mater. Chem. A.

[39] Robeson L.M., Dose M.E., Freeman B.D., Paul D.R. (2017). Analysis of the transport properties of thermally rearranged (TR) polymers and polymers of intrinsic microporosity (PIM) relative to upper bound performance. J. Membr. Sci..

[40] Ghanem B.S., Swaidan R., Litwiller E., Pinnau I. (2014). Ultra-microporous triptycene-based polyimide membranes for high-performance gas separation. Adv. Mater..

[41] Ma X., Pinnau I. (2016). A novel intrinsically microporous ladder polymer and copolymers derived from 1,1′,2,2′-tetrahydroxy-tetraphenylethylene for membrane-based gas separation. Polym. Chem..

[42] Bezzu C.G., Carta M., Ferrari M.C., Jansen J.C., Monteleone M., Esposito E., Fuoco A., Hart K., Liyana-Arachchi T.P., Colina C.M. (2018). The synthesis, chain-packing simulation and long-term gas permeability of highly selective spirobifluorene-based polymers of intrinsic microporosity. J. Mater. Chem. A.

[43] Ghanem B.S., McKeown N.B., Budd P.M., Al-Harbi N.M., Fritsch D., Heinrich K., Starannikova L., Tokarev A., Yampolskii Y. (2009). Synthesis, characterization, and gas permeation properties of a novel group of polymers with intrinsic microporosity: PIM-polyimides. Macromolecules.

[44] Alentiev D.A., Egorova E.S., Bermeshev M.V., Starannikova L.E., Topchiy M.A., Asachenko A.F., Gribanov P.S., Nechaev M.S., Yampolskii Y.P., Finkelshtein E.S. (2018). Janus tricyclononene polymers bearing tri(*n*-alkoxy)silyl side groups for membrane gas separation. J. Mater. Chem. A.

