# Supplementary material for: Polyvinylnorbornene Gas Separation Membranes
Source: Polymers (Basel). 2019 Apr 17;11(4):704. doi: 10.3390/polym11040704 (PMC6523562; doi:10.3390/polym11040704)
Supplement: Supplementary file 1 [file polymers-11-00704-s001.pdf]

Supporting information

Polyvinylnorbornene gas separation membranes

Wouter Dujardin[1,2], Cédric Van Goethem[2], Julian A. Steele[2], Maarten Roeffaers[2], Ivo F.J. Vankelecom[2], Guy Koeckelberghs[1*]

[1] Laboratory for Polymer Synthesis, Department of Chemistry, KU Leuven, Celestijnenlaan 200F, B-3001 Heverlee, Belgium; wouter.dujardin@kuleuven.be, guy.koeckelberghs@kuleuven.be
[2] Centre for Surface Chemistry and Catalysis, Department of Microbial and Molecular Systems, KU Leuven, Celestijnenlaan 200F, B-3001 Heverlee, Belgium; cedric.vangoethem@kuleuven.be, Julian.steele@kuleuven.be, maarten.roeffaers@kuleuven.be, ivo.vankelecom@kuleuven.be
* Correspondence: guy.koeckelberghs@kuleuven.be

GPC

Table S1. GPC data of homopolymers pNB and pVNB and copolymers pNB-VNB-50, prepared by different catalyst systems.

Entry #	Polymer	Catalyst system	$\frac{[monomer]_0}{[catalyst]_0}$	$\bar{M}_n$ (kg/mol)	$\bar{M}_w$ (kg/mol)	Đ
1	**pNB**	$Ni(C_6F_5)_2(SbPh_3)_2$	1000	84	277	3.3
2	pNB-VNB-50	$Ni(C_6F_5)_2(SbPh_3)_2$	1000	26	61	2.3
3	pNB-VNB-50	$Pd_2dba_3/AgSbF_6/PPh_3$	2000	12	52	4.5
4	**pNB-VNB-50**	Pd_2dba_3/TTPB/PCy_3	1000	167	440	2.6
5	pVNB	$Ni(C_6F_5)_2(SbPh_3)_2$	1000	11	23	2.0
6	pVNB	$Pd_2dba_3/AgSbF_6/PPh_3$	5000	5	19	3.5
7	**pVNB**	Pd_2dba_3/TTPB/PCy_3	1000	250	400	1.6

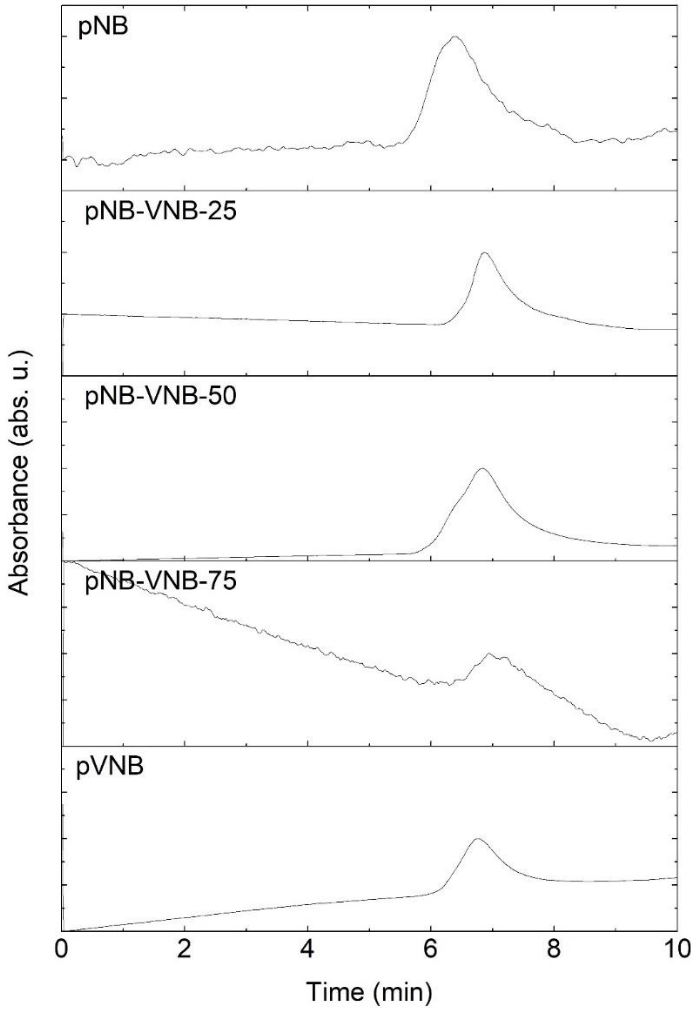

Figure S1. GPC spectra of polynorbornenes with increasing vinyl content.

FT-IR

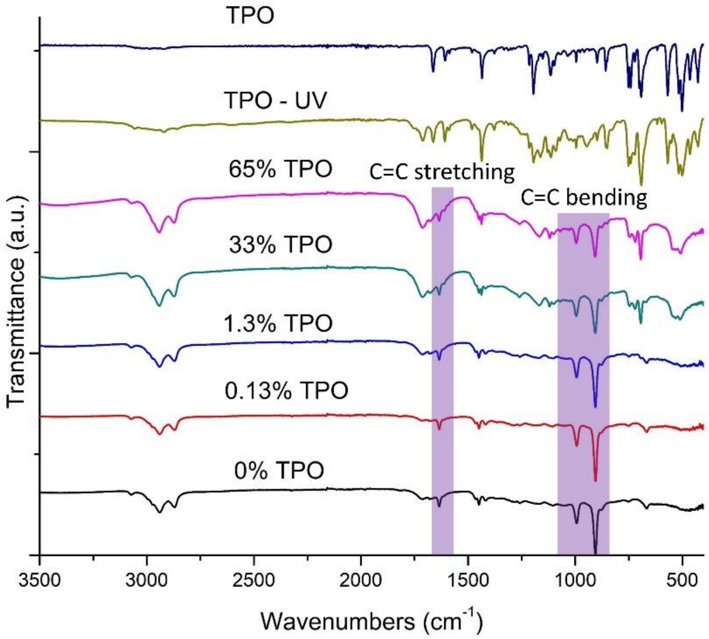

Figure S2. FT-IR of pVNB with increasing TPO loading. 'TPO' and 'TPO - UV' are, respectively, unexposed and exposed TPO to UV light.

TGA

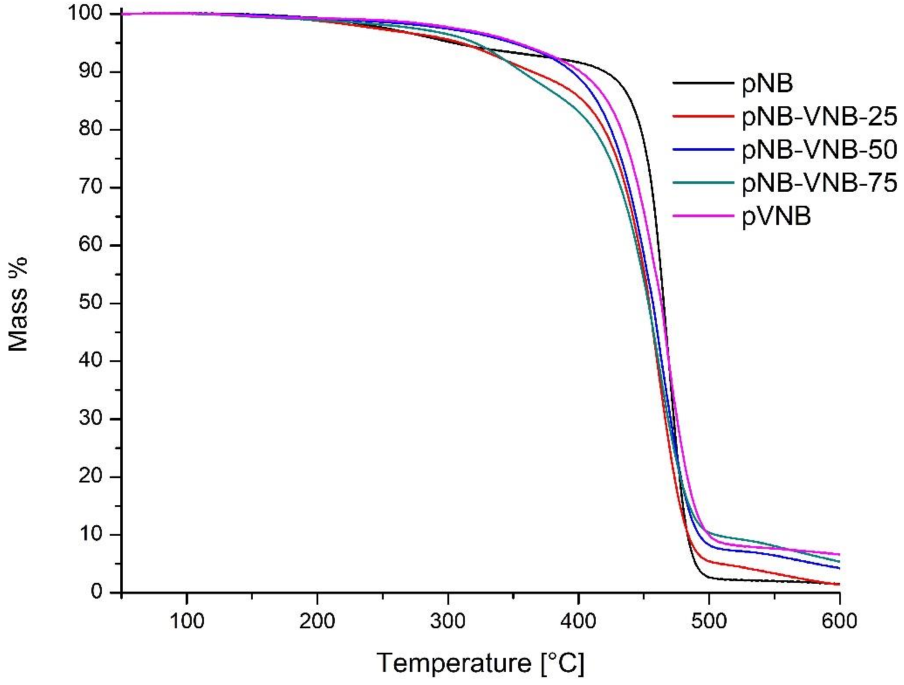

Figure S3. TGA thermogram of polynorbornenes membranes with increasing VNB content.

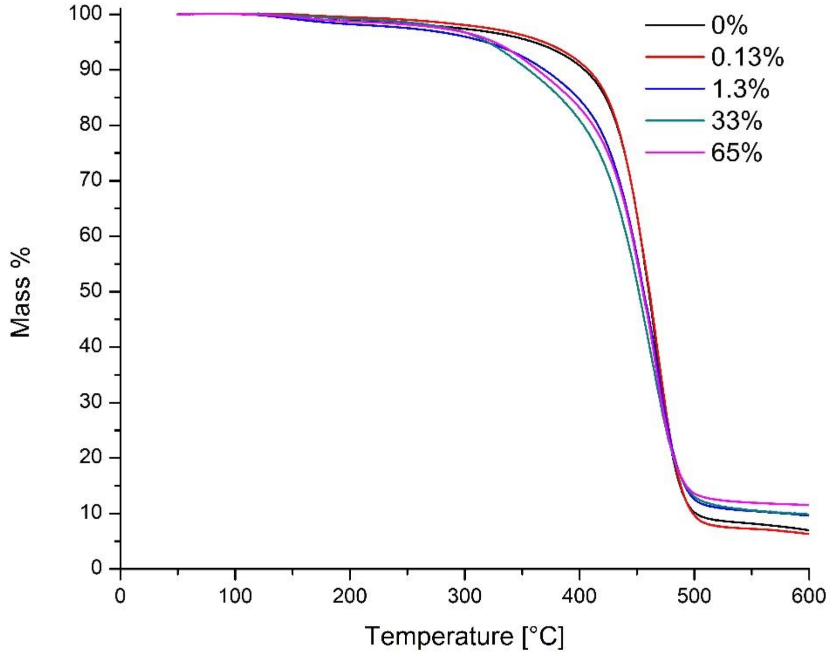

Figure S4. TGA thermogram of crosslinked polyvinylnorbornene membranes.

DSC

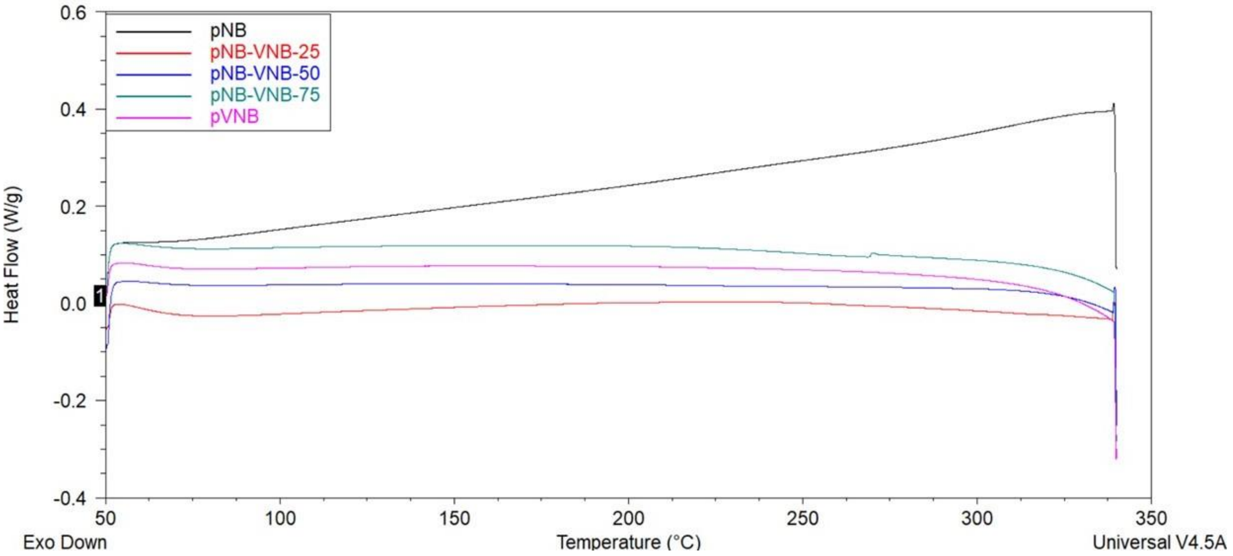

Figure S5. DSC of polynorbornenes with increasing VNB content.

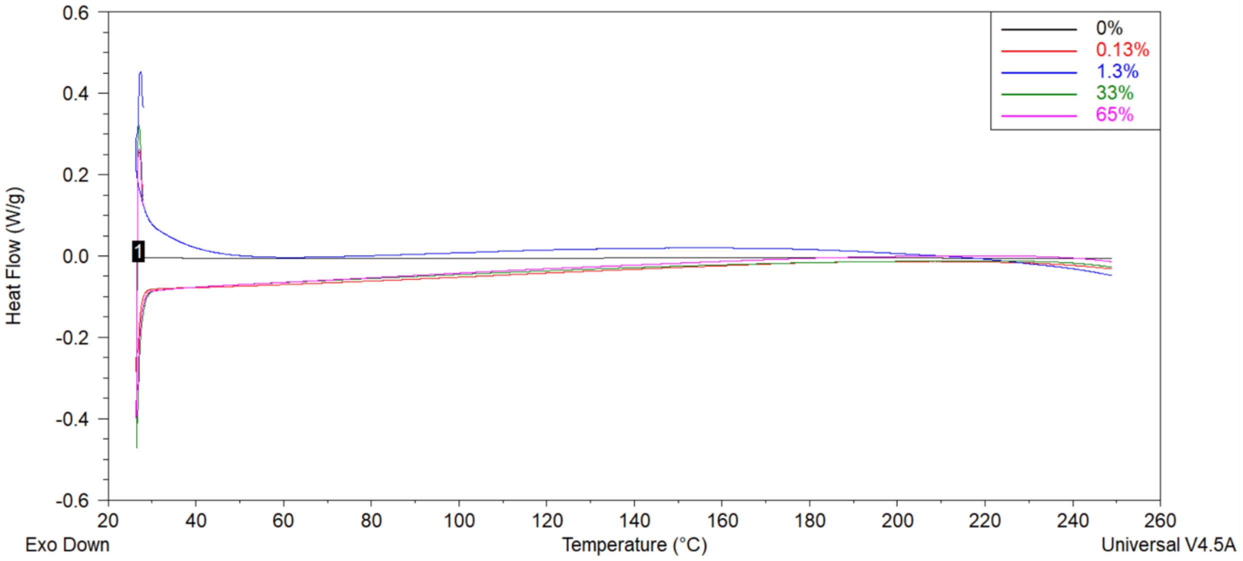

Figure S6. DSC of polynorbornenes with increasing TPO loading.

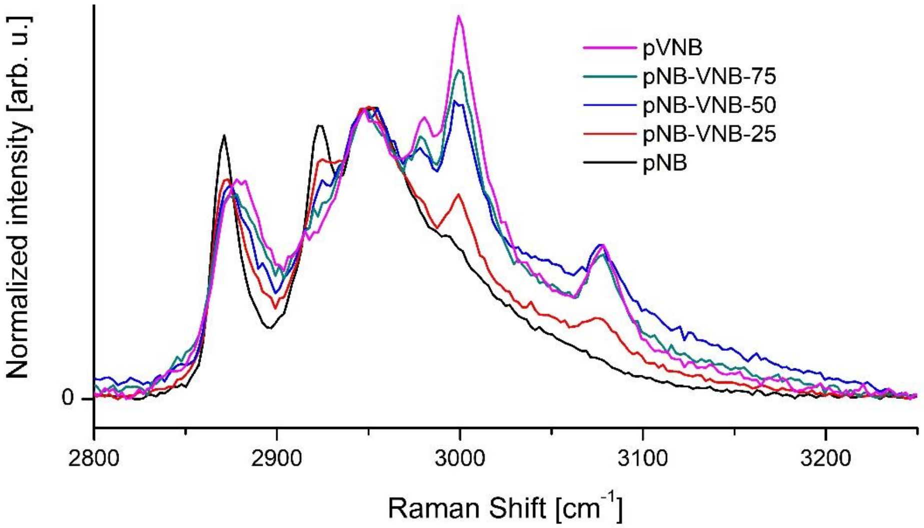

Figure S7. Comparison of Raman spectra recorded over the CH_x stretching region, for rising VNB in monomer feed. These spectra have been normalized relative to their common band at 2950 cm^{-1}, which does not evolve with changing VNB concentration, allowing for the C=C band analysis shown in Figure 4 of the main article.

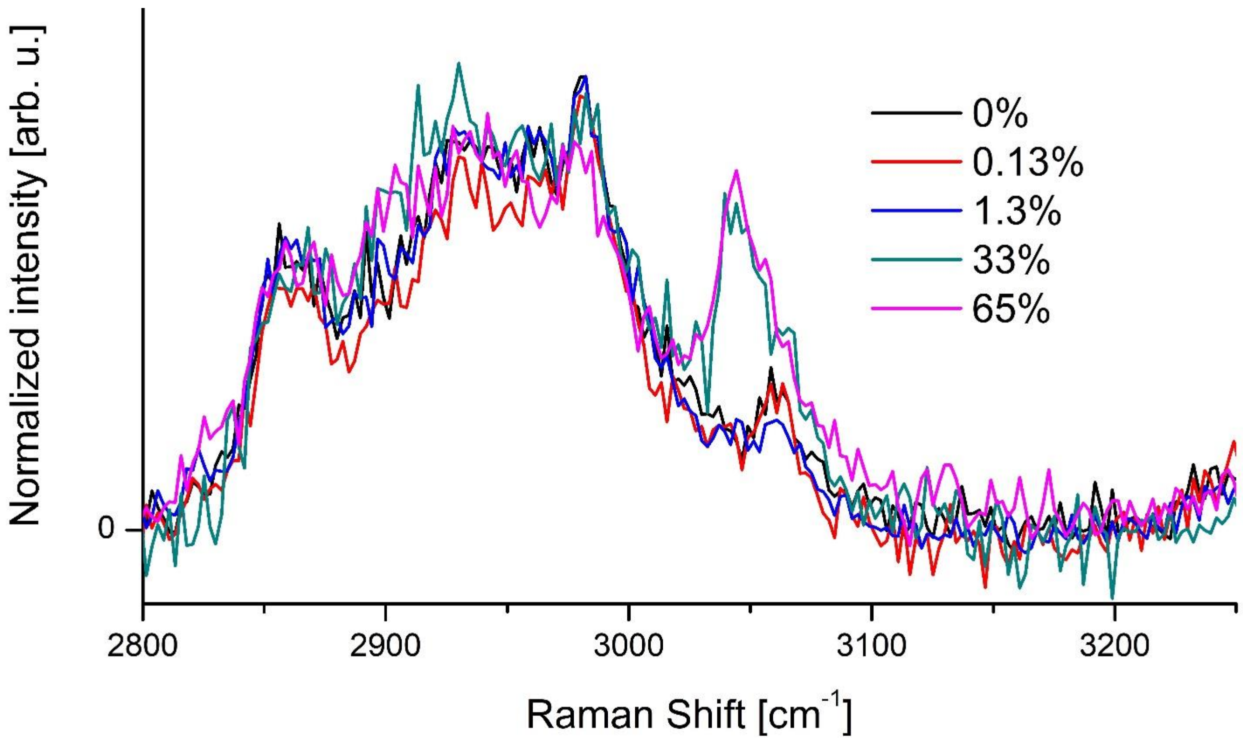

Figure S8 Comparison of Raman spectra recorded over the CH_x stretching region, for increasing TPO content in the pVNB membranes. These spectra have been normalized relative to their common band at 2950 cm^{-1}, which does not evolve with changing VNB concentration, allowing for the C=C band analysis shown in Figure 9 of the main article.

SEM

pNB

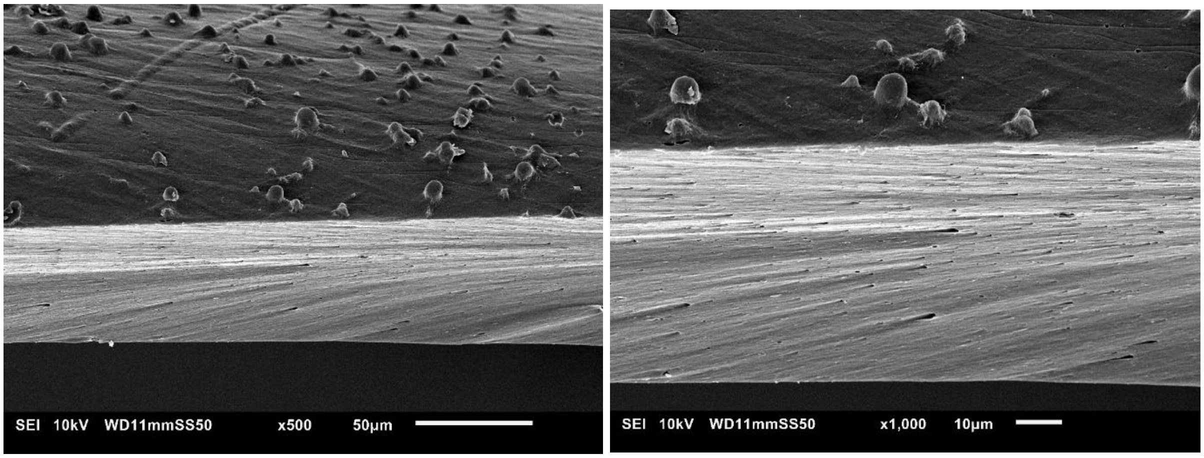

pNB-VNB-25

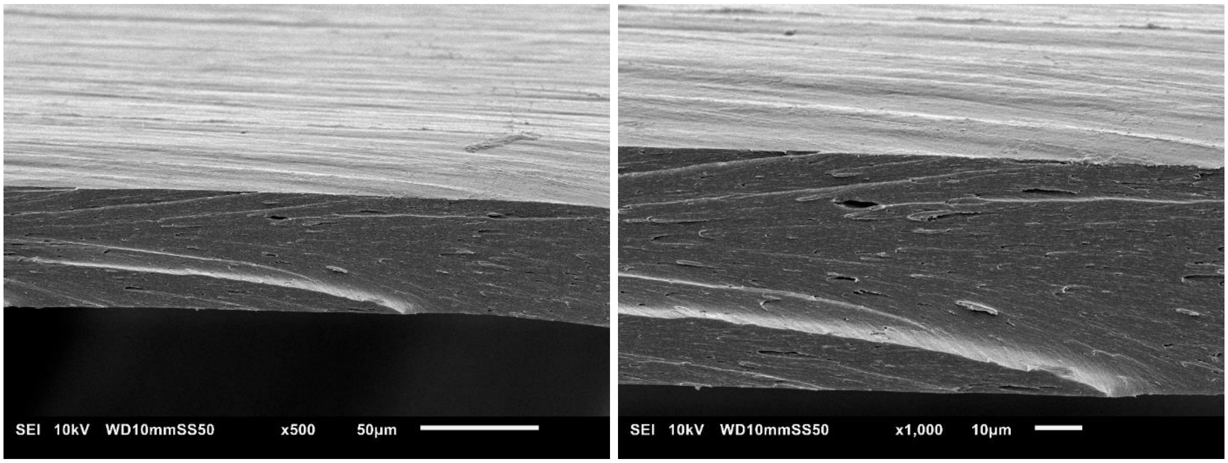

pNB-VNB-50

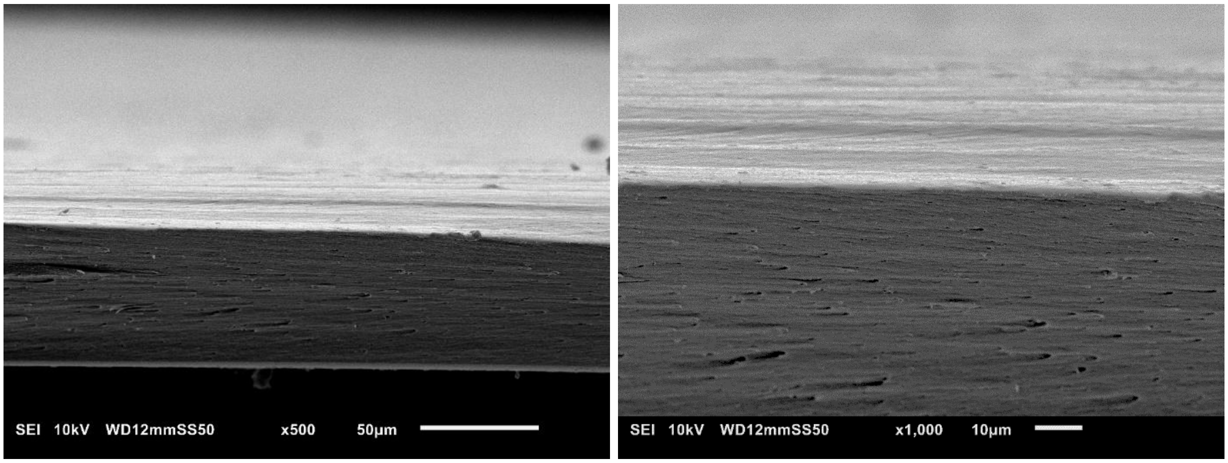

pNB-VNB-75

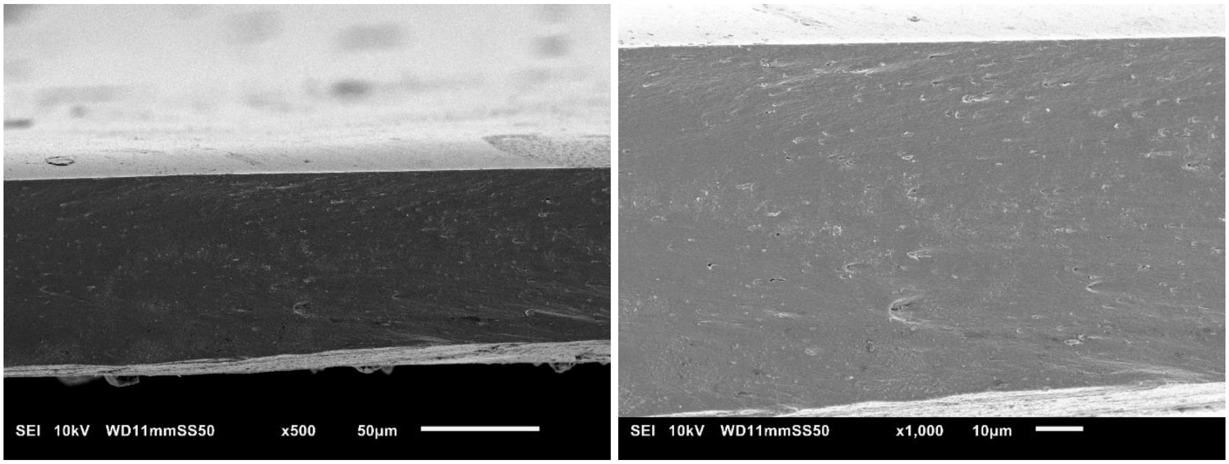

pVNB

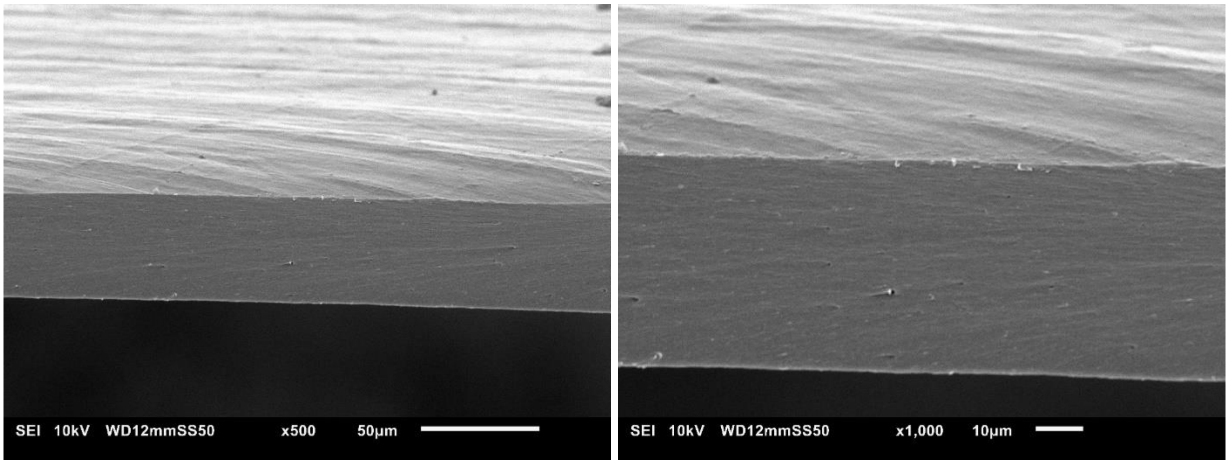

Figure S9. SEM images of polynorbornene copolymers with increasing VNB content.

NMR

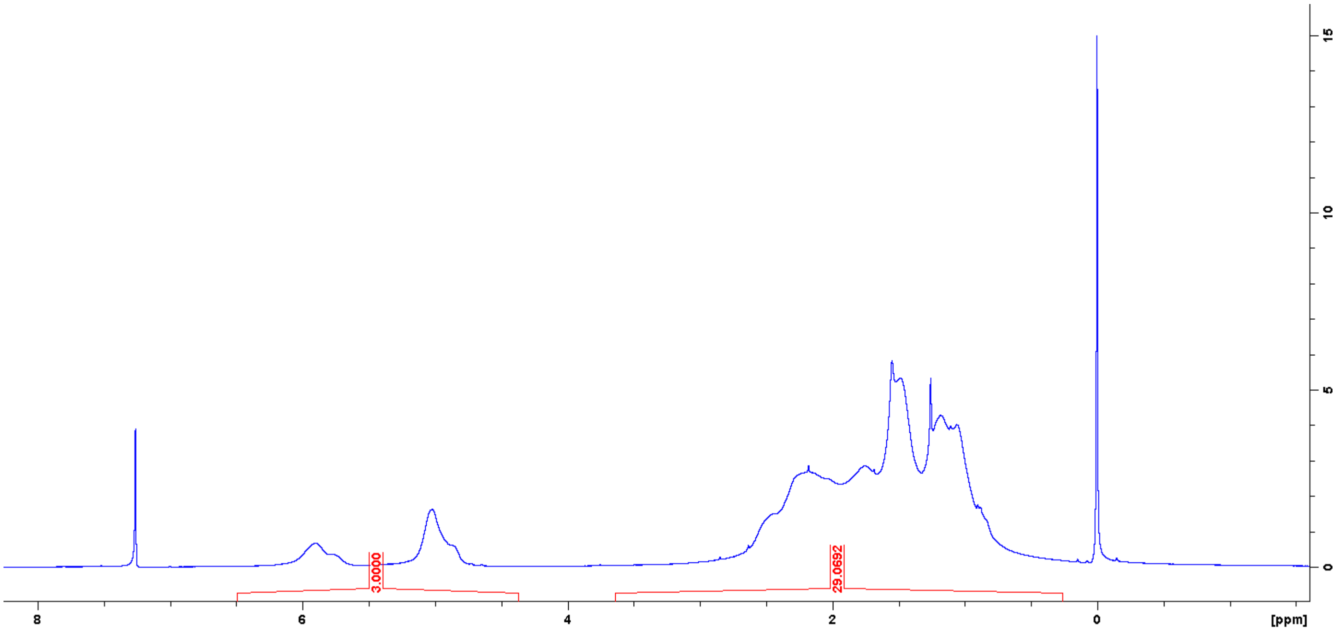

Figure S10. ^{1}H NMR spectrum of pNB-VNB-25. (400 MHz, $CDCl_3$)

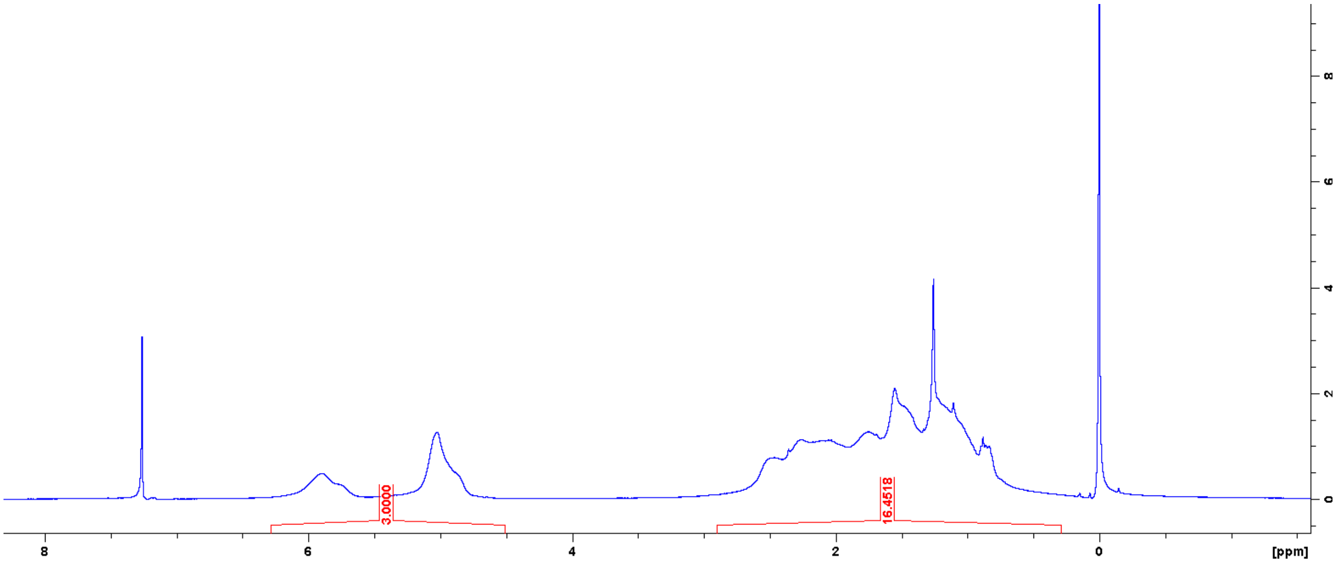

Figure S11. ^{1}H NMR spectrum of pNB-VNB-50. (400 MHz, $CDCl_3$)

The ratio of aliphatic to vinyl signals with pVNB is 9:3. Therefore, the following formula can be used to calculate the VNB content in the copolymers:

$$\text{VNB content} = \frac{9}{\text{integration of aliphatic signal}} \times 100\%$$

For pNB-VNB-25, this gives 31%. For pNB-VNB-50, this gives 54%.
